# Defining Time in Acute Upper Gastrointestinal Bleeding: When Should We Start the Clock?

**DOI:** 10.3390/jcm12072542

**Published:** 2023-03-28

**Authors:** Riccardo Marmo, Marco Soncini, Cristina Bucci, Clelia Marmo, Maria Elena Riccioni, on behalf of the GISED Study Group

**Affiliations:** 1 Gastroenterology and Endoscopy Unit, “L. Curto” Hospital Polla, ASL Salerno, 84025 Polla, Italy; 2 Department of Internal Medicine, “A. Manzoni” Hospital, 23900 Lecco, Italy; 3 Gastroenterology and Hepatology Unit, AORN Santobono-Pausillipon, 80129 Napoli, Italy; 4 Endoscopia Digestiva Chirurgica, Fondazione Policlinico Universitario A. Gemelli, IRCCS, Università Cattolica del Sacro Cuore, 00168 Rome, Italy

**Keywords:** acute gastrointestinal bleeding, scoring risk, mortality, prediction, timing to endoscopy

## Abstract

Introduction: The execution of upper endoscopy at the proper time is key to correctly managing patients with upper gastrointestinal bleeding (UGIB). Nonetheless, the definition of “time” for endoscopic examinations in UGIB patients is imprecise. The primary aim of this study was to verify whether the different definitions of “time” (i.e., the symptoms-to-endoscopy and presentation-to-endoscopy timeframes) impact mortality. The secondary purpose of this study was to evaluate the similarity between the two timeframes. Methods: A post-hoc analysis was performed on a prospective multicenter cohort study, which included UGIB patients admitted to 50 Italian hospitals. We collected the timings from symptoms and presentation to endoscopy, together with other demographic, organizational and clinical data and outcomes. Results: Out of the 3324 patients in the cohort, complete time data were available for 3166 patients. A significant difference of 9.2 h (*p* < 0.001) was found between the symptoms-to-endoscopy vs. presentation-to-endoscopy timeframes. The symptoms-to-endoscopy timeframe demonstrated (1) a different death risk profile and (2) a statistically significant improvement in the prediction of mortality risk compared to the presentation-to-endoscopy timeframe (*p* < 0.0002). The similarity between the two different timeframes was moderate (K = 0.42 ± 0.01; *p* < 0.001). Conclusions: The symptoms-to-endoscopy and presentation-to-endoscopy timeframes referred to different timings during the management of upper endoscopy in bleeding patients, with the former being more accurate in correctly identifying the mortality risk of these patients. We suggest that further studies be conducted to validate our observations, and, if confirmed, a different definition of time should be adopted in endoscopy.

## 1. Introduction

It is well known that the execution of upper endoscopy at the proper time is key to correctly managing patients with upper gastrointestinal bleeding (UGIB) [1]. The timing of endoscopic examinations not only has an impact on clinical outcomes (i.e., mortality, re-bleeding, need for surgery, etc.) but also hospital organization (i.e., 24/7 service vs. day service, etc.), healthcare costs (i.e., length of hospital stay) and legal aspects [2,3,4,5]. Nonetheless, the definition of “time” for endoscopic examinations in UGIB patients is still imprecise.

Two different timeframes are used when endoscopic examinations are performed on patients with UGIB: the first is defined as the time from the onset of symptoms to emergency department (ED) admission; the second is defined as the time from emergency department admission to endoscopy. By convention, we are used to considering the “time before endoscopy” timeframe as the time from emergency department admission to the endoscopy, taking no notice of what happened before. In 2021, the ESGE (European Society of Gastrointestinal Endoscopy) recommended that following hemodynamic resuscitation, early upper endoscopy should be performed in patients with non-variceal bleeding (≤24 h from when the patient arrives at the ED) [6]. In 2021, the ACG (American College of Gastroenterology) suggested that patients undergo upper endoscopy within 24 h of presentation, adding other timeframes from patient presentation to triage to hospital admission, considering that in large hospitals, admission may not necessarily coincide with the patient’s arrival [7]. Lastly, in a large randomized clinical trial in 2020, Lau et al. defined the “time” as the interval between gastroenterologist consultation and upper endoscopy. They showed no effect of early endoscopy (<6 h) on mortality and re-bleeding compared to the 6–24 h group [3]. In 2011, we showed that very early (≤6 h) or early (≤12 h) endoscopy was associated with an increase in mortality rate in high-risk patients [8]. Consequently, using one definition or another is not generalizable and may lead to clinical misevaluations or legal implications in the case of worrying outcomes. The primary aim of this study was to verify whether the two definitions of “time” impact mortality in patients with upper gastrointestinal bleeding. The secondary aim was to evaluate the similarity between the two following timeframes: from symptoms onset to upper endoscopy and from ED presentation to upper endoscopy.

## 2. Materials and Methods

This study was conducted as a nationwide cohort study based on data derived from the GISED (Gruppo Italiano Studio Emorragia Digestiva) Study Group database, which prospectively collected records on all patients admitted for acute UGIB from 1 January 2014 to 31 December 2015 in 50 Italian hospitals. Only patients with ongoing bleeding and endoscopically documented UGIB were considered eligible. The patients’ clinical features, basic lab tests, clinical evolution during hospitalization, procedures, therapies, outcomes and comorbidities were recorded during the study period. Data on the time elapsed from symptom onset to endoscopy and from ED presentation to endoscopy were also recorded. Upper gastrointestinal bleeding was defined by the presence of overt ongoing upper gastrointestinal hemorrhage or hematemesis/coffee grounds vomiting, melena, syncope or a combination of the above, as determined by upper endoscopy. All patients were scheduled to receive upper endoscopy within 24 h [6]. During endoscopy, UGIB was classified as variceal or non-variceal and Forrest’s classification was used to classify the types of bleeding lesions as required. Shock was defined using the shock index (≥1). Re-bleeding was defined as a new episode of hematemesis > 6 h after endoscopy, the presence of melena or hematochezia after the normalization of feces color, tachycardia (heart rate > 110/min) or hypotension (systolic arterial pressure < 90 mmHg) in the absence of any other plausible cause, a hemoglobin (Hb) decrease > 2 gr/dL following two stable Hb values or a Hb decrease > 3 gr/dL associated with melena or hematochezia within 24 h, as confirmed by endoscopy [9]. Bleeding-related all-cause mortality was defined when death occurred within 30 days for patients with non-variceal bleeding, and 42 days for those with variceal bleeding [9]. Bleeding severity was defined as the presence of at least one of following conditions: systolic blood pressure < 90; pulse rate ≥ 100; and hemoglobin levels < 10 gr/L [10]. Patients were defined as needing treatment when they had a blood transfusion or any operative or endoscopic intervention to control their bleeding, or had undergone no interventions but had died after admission. The presence of comorbidities was classified according to the Charlson comorbidity index, and the performance status was classified according to the American Society of Anesthesiologist physical status classification system (ASA score) at admission [11,12]. Data needed for full Rockall, PNED, AIMS65 and Glasgow-Blatchford scores were also collected to calculate the ABC score [13,14,15,16]. Patients were excluded when lower or intermediate gastrointestinal bleeding was clinically suspected or they were unavailable to express informed consent to the data collection. To evaluate the clinical outcomes, physicians not involved in the endoscopic procedure or local research nurses followed up with patients for 30 days after hospital admission for non-variceal hemorrhage, or 42 days for variceal bleeding. All-cause mortality was defined as when death occurred within 30 or 42 days. For outpatients, two endoscopy timeframes were identified: the time from the onset of symptoms to endoscopy (symptoms-to-endoscopy group); and the time from hospital presentation (i.e., arrival at the emergency department) to endoscopy (presentation-to-endoscopy group). For inpatient bleeding patients, the symptoms-to-endoscopy timeframe was defined as the time elapsed from the onset of symptoms to upper endoscopy, and the presentation-to-endoscopy timeframe was defined as the time elapsed from gastroenterologist consultation to upper endoscopy. The timeframe before the endoscopy procedure was divided into ≤6 h, 6–12 h, 12–24 h and ≥24 h. The time was expressed as hours and minutes (for example, 17.1 h = 17 h and 10 min). Impact on mortality was assessed using the mortality prediction performance of the two timeframes. The Institutional Review Board in each participating center approved the study protocol, following Ethical Committee approval (N. 556, 26 June 2013; San Carlo Borromeo Hospital, Milan). Written informed consent was obtained from all patients or their healthcare proxies.

### Statistical Analysis

Assuming that the area under the receiver operating characteristic (AUROC) curve was 0.51 for mortality in the presentation-to-endoscopy group, and at least 10% more in the symptoms-to-endoscopy group, with an α error of 5%, a power of 90%, a rank correlation of 0.4 and a mortality rate of 5.4% [17], we determined that we needed a sample of 2570 patients. The selected parameters were analyzed using appropriate descriptive statistics (i.e., mean, standard deviation (SD), median, interquartile range, proportion and 95% confidence interval (C.I.)). A comparison of the continuous variables was performed using an appropriate one-way analysis of variance. The proportions were compared using chi-squared and Fisher’s exact tests, as appropriate. As we assumed that the symptoms-to-endoscopy and presentation-to-endoscopy times were predictive factors for mortality, they were included in a multiple logistic regression model to evaluate their independent role. The results were presented as odds ratios (ORs) with 95% confidence intervals (C.I.s). An AUROC with a 95% C.I. was derived using the routine L-ROC to evaluate the discriminative performance of mortality prediction. The two performance ROC curves were subsequently compared to establish the similarity between the two ROC areas using the DeLong method [18]. All statistical analyses were performed using STATA software, version 15.1 (StataCorpLp, College Station, TX, USA), according to the transparent reporting of a multivariable prediction model for individual prognosis or diagnosis (TRIPOD) statement. 

## 3. Results

During the study period, 3324 UGIB patients were enrolled. The bleeding source was determined to be non-variceal in 2764 patients and variceal in 560. Out of the 3324 patients, complete time data were available for 3166 patients.

### 3.1. Comparison between Timeframes

Death occurred in 209/3166 patients (6.6%). The demographics, descriptive data and significant outcomes of all patients (both in- and outpatients) are shown in Table 1. For all patients (overall population), the mean time lapsed from the onset of symptoms to endoscopy was 17.3 h (95% C.I. = 17–18.1 h), while the mean time lapsed from presentation to endoscopy was 8.5 h (95% C.I. = 8.1–9.3 h), with a statistically significant difference of 9.2 h (95% C.I. = 8.3–10.1 h; *p* < 0.001). The demographics, baseline characteristics and outcomes of the patients in the two timeframe groups are shown in the Supplementary Materials (Tables S1–S8).

Splitting the population between in- and outpatients, we found that the difference was driven mainly by the outpatient population (symptoms-to-endoscopy time = 18.1 h vs. presentation-to-endoscopy = 8.2 h; mean = 10.3 h; *p* < 0.001), as there were no differences between the two timeframes among the inpatients (13.5 h vs. 12.2 h; mean = 1.3 h; *p* = 0.25). The similarity between the different timeframes was then evaluated. The data for all patients in the two groups are provided in the Supplementary Materials (Tables S9 and S10).

As shown in Table 2, the agreement between the two timeframes was moderate (K = 0.42 ± 0.01; *p* < 0.001), which could be explained by cross verifying the number of patients for each timeframe. Indeed, roughly one third of the population received an upper endoscopy less than 6 h from the onset of symptoms (1229 patients), with a mortality rate of 8.7% (107/1229), while the rest were spread across the other timeframes. Interestingly, of the 2072 patients who received an endoscopy ≤ 6 h from hospital presentation, 243 patients had symptoms start ≥ 24 h before the endoscopic examination, thereby confirming the broad variability in the evaluation of the “right” time for endoscopy (Table 2). Analyzing these 243 patients, we found that 77% had an upper endoscopy during the day service (from 8 am to 8 pm), 87% were non-cirrhotic patients presenting with melena (82%) and their mortality rate was 4.5%. These numbers were similar in the 130 patients whose symptoms started ≥ 24 h before and received an upper endoscopy ≥ 24 h after hospital presentation. Of these, 91% had an upper endoscopy during the day service (from 8 am to 8 pm), 91% were non-cirrhotic patients presenting with melena (66%) and their mortality rate was 3.8%.

### 3.2. Mortality Prediction

**Figure 1:**
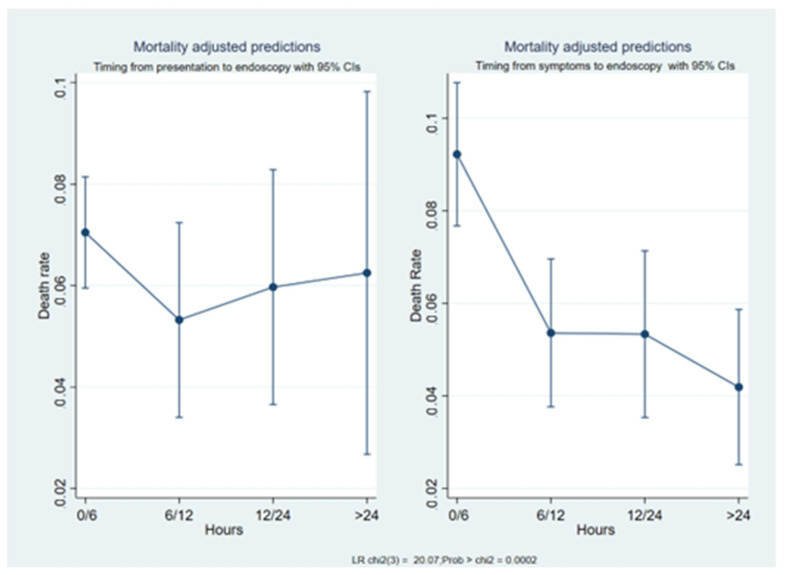
The mortality prediction according to the two timeframes.

**Figure 2:**
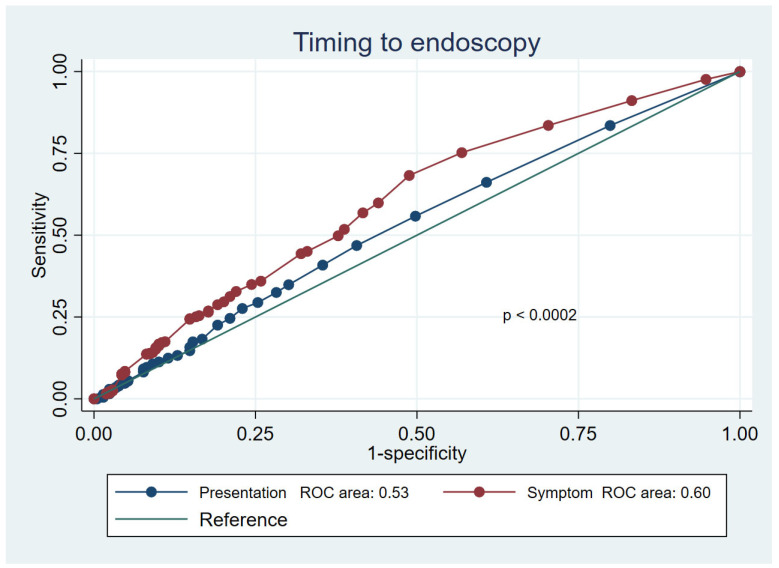
The differences in the prediction of mortality risk between the two timeframes.

The median value of the most common mortality prediction score was significantly lower (*p* ≤ 0.001) in the symptoms-to-endoscopy group than in the presentation-to-endoscopy group, both within the overall cohort study and the outpatients subset (Tables S1, S3 and S5 in the Supplementary Materials). Comparing the mortality rates of the different subgroups of patients, we found that the difference was statistically significant between: the very early admission (0–6 h) and very early endoscopy (0–6 h) group (1229 patients); and the very early endoscopy and late symptoms onset (≥24 h) group (243 patients) (8.7% vs. 4.5%; O.R. = 0.49; 95% C.I. = 0.26–0.93; *p* < 0.03); and the late endoscopy (≥24 h) and late symptoms onset (≥24 h) group (130 patients) (8.7% vs. 3.8%; O.R. = 42; 95% C.I. = 0.17–1.01; *p* < 0.05) (Table 2). We also defined the mortality risk according to the different timeframes. In the presentation-to-endoscopy group, the death risk had a not-statistically-significant decreasing trend in patients who received an endoscopy in 0–6 h compared to those who received an endoscopy later (Table 3). In this group, the death risk showed a “U” shape curve and the overall difference between the timeframes was not significant (O.R. = 1.13; *p* = 0.17) (Figure 1). In the symptoms-to-endoscopy group, mortality significantly decreased as the time increased (Table 4 and Figure 1) (overall O.R. = 0.71; *p* < 0.001). As a consequence, the difference between the mortality prediction performance of the two timeframes was statistically significant (*p* < 0.002), with the symptoms-to-endoscopy timeframe being more accurate (Figure 2). According to our multivariate logistic regression (Table 5), the symptoms-to-endoscopy timeframe confirmed its independent and significant role as a death risk factor, depicting and demonstrating a higher accuracy than the presentation-to-endoscopy timeframe, independently of the performance status and the bleeding severity.

## 4. Discussion

In recent years, many researchers have focused on defining the best time to perform upper endoscopy in bleeding patients. Endoscopy should not be performed after 24 h, but performing the procedure earlier does not necessarily produce better outcomes [2,3,8]. The researchers who have explored this topic have used different time to endoscopy definitions, so no general consensus exists. Lau [2,3] defined the time to endoscopy as the timeframe between gastroenterologist consultation and endoscopy. Laursen [18], in a recent observational study, defined the time as the timeframe from hospital admission (i.e., arrival at the emergency department) to endoscopy. Our study defined the time to endoscopy in patients with acute upper GI bleeding by combining the most common definitions, i.e., the time from the onset of symptoms to endoscopy and the time from hospital presentation to endoscopy. We found that in our cohort, the two timeframes were significantly different (mean = 9.2 h; *p* <0.001), implying distinct death risks and different approaches to bleeding patients. In the presentation-to-endoscopy group, the mortality rate showed a U-shaped curve (similar to that reported by Laursen et al. [19]) and was higher in patients who underwent endoscopy within 6 h or after 24 h from hospital presentation and lower in those who received an endoscopy between 6 and 24 h from hospital admission. Additionally, when we only considered the death risk among patients who underwent very early endoscopy (i.e., less than 6 h from hospital presentation), we found a difference in mortality rate between those whose symptoms started <6 h or ≥24 h (8.7% vs. 4.5%). Patients with different score values (GB, ABC, AIMS65, PNED and Rockall scores) were grouped in the same presentation-to-endoscopy timeframe more frequently than those in the symptoms-to-endoscopy group. The presentation-to-endoscopy timeframe could reflect hospital performance more than the actual clinical need. Conversely, in the symptoms-to-endoscopy group, we observed that the longer the time from symptoms onset to upper endoscopy, the lower the mortality rate. Moreover, patients with less severe clinical conditions (based on ASA or bleeding scores) underwent endoscopy at the right time more frequently (i.e., they were more frequently allocated the right time slot), possibly reflecting that clinical assessments were not influenced by organizational need, making this timeframe more appropriate for the global judgment of patients. The strengths of this study were that it was the first study to analyze and define the concept of “time” in the management of patients with upper gastrointestinal bleeding. Secondly, the study was conducted in local and third-level hospitals spread throughout Italy, including many patients with both variceal and non-variceal bleeding. The limitations of this study were that we were unable to differentiate between hospital admission, hospital presentation and gastroenterology consultation. We also found that the definition of symptom onset was imprecise as it depended on the patient’s recollection; however, the difference in the presentation-to-endoscopy timeframe was so significant (9 h) that minor deviations would not have changed our results. Additionally, this cohort considered patients who were submitted for endoscopy because of ongoing bleeding. Patients with previous overt bleeding were not considered in the present analysis as they did not need urgent endoscopy.

### Perspective

Clinical performance status and hemodynamic conditions should dictate when to perform endoscopy in patients with ongoing or overt bleeding. In patients without any comorbidities, the time to endoscopy as it is currently defined does not impact survival [18]. However, the time to endoscopy should be more appropriately redefined, according to our data. Our findings were based on the physician’s point of view (i.e., a hospital performance measure) more than the actual endoscopy needs of patients, which could be regarded as the central aspect that impacts survival. Future studies should use both definitions to evaluate the best time to perform endoscopy.

## 5. Conclusions

In conclusion, the symptoms-to-endoscopy and presentation-to-endoscopy timeframes referred to different timings while managing patients with bleeding during upper endoscopy, with the former being more accurate for correctly identifying the mortality risk of these patients. We suggest that further studies should validate our observations and, if confirmed, a different definition of time should be adopted in endoscopy.

## Figures and Tables

**Table 1:** The baseline features of the cohort.

	*n* = 3166
Age, mean [± SD]	68.2 [±15.7]
Male gender, *n* (%)	2137 (67.5)
Hemodynamic instability, yes, *n* (%)	226 (7.1)
Outpatients, *n* (%)	2672 (84.4)
**Bleeding source, *n* (%)**	
Non-variceal	2632 (83.1)
Variceal	534 (16.9)
**ASA score, *n* (%)**	
I	842 (26.6)
II	1100 (34.7)
III	1029 (32.5)
IV	195 (6.2)
**“Presentation-to-Endoscopy” timeframe, number of patients (%)**	
0–6 h	2072 (65.4)
6–12 h	520 (16.4)
12–24 h	399 (12.6)
≥24 h	175 (5.5)
**“Symptoms-to-Endoscopy” timeframe, number of patients (%)**	
0–6 h	1304 (41.2)
6–12 h	745 (23.5)
12–24 h	578 (18.3)
≥24 h	539 (17)
Rockall score, mean (95% C.I.)	4 (3–5)
Glasgow-Blatchford score, mean (95% C.I.)	7 (5–9)
ABC score, mean (95% C.I.)	5 (3–6)
AIMS65 score, mean (95% C.I.)	1 (1–2)
PNED score, mean (95% C.I.)	3 (1–6)
Endoscopy performed	
On call, number (%)	904 (28.5)
Weekend, number (%)	748 (23.6)
**Clinical outcomes**	
Transfusion, yes (%)	1885 (59.5)
Re-bleeding, yes (%)	192 (6.1)
Need for interventional radiology, yes (%)	30 (0.9)
Need for surgery, yes (%)	102 (3.2)
Mortality, yes (%)	209 (6.6)
Length of stay, days [±SD]	9.5 [±9]

SD, standard deviation; C.I., confidence interval; ASA score, American Society of Anesthesiologist physical status classification system; GB, Glasgow-Blatchford score; ABC score, age, blood tests and comorbidities score; AIMS65, albumin levels less than 3.0 g/dL, INR levels greater than >1.5, altered mental status, systolic blood pressure 90 mm Hg or lower and older than 65 years; PNED, Progetto Nazionale Endoscopia Digestiva.

**Table 2:** A comparison between the two timeframes within the overall cohort (expressed as the number of patients).

Symptoms-to-Endoscopy Group	Presentation-to-Endoscopy Group				
	0–6 h	6–12 h	12–24 h	≥24 h	Total
0–6 h	1229	22	28	25	1304
6–12 h	405	307	26	7	745
12–24 h	195	145	225	13	578
≥24 h	243	46	120	130	539
Total	2072	520	399	175	3166

Agreement = 78.03%; appa = 0.42; SE = 0.01. Expected agreement = 62%; Z = 35.46; *p* < 0.001.

**Table 3:** The univariate mortality risk according to presentation-to-endoscopy timeframes.

Timeframe	Odds Ratio	z	*p* > |z|	95% Confidence Interval
0–6 h	Reference			
6–12 h	0.75	−1.35	0.177	0.50–1.14
12–24 h	0.84	−0.74	0.457	0.54–1.32
≥24 h	0.88	−0.38	0.705	0.47–1.67
_cons	0.08	−30.05	0.000	0.06–0.09

**Table 4:** The univariate mortality risk according to the symptoms-to-endoscopy timeframes.

Timeframe	Odds Ratio	z	*p* > |z|	95% Confidence Interval
0–6 h	Reference			
6–12 h	0.55	−3.15	0.002	0.37–0.80
12–24 h	0.57	−2.65	0.008	0.38–0.87
≥24 h	0.45	−3.39	0.001	0.29–0.72
_cons	0.10	−23.91	0.000	0.08–0.12

**Table 5:** The multivariate mortality risk according to the two sets of timeframes.

Timeframe	Odds Ratio	z	*p* > |z|	95% Confidence Interval
Symptoms-to-endoscopy				
0–6 h	Reference			
6–12 h	0.62	−2.08	0.038	0.40–0.97
12–24 h	0.60	−1.97	0.049	0.36–1.00
≥24 h	0.46	−2.80	0.005	0.27–0.79
Presentation-to-endoscopy				
0–6 h	Reference			
6–12 h	0.94	−0.25	0.802	0.56–1.56
12–24 h	1.06	0.19	0.848	0.61–1.84
≥24 h	1.20	0.49	0.623	0.58–2.49
Bleeding severity	1.76	2.50	0.013	1.13–2.74
ASA score				
ASA I	Reference			
ASA II	4.34	3.81	0.000	2.04–9.26
ASA III	8.60	5.76	0.000	4.13–17.89
ASA IV	47.98	9.97	0.000	22.42–102.69

## Data Availability

The data presented in this study are available upon request from the corresponding author. The data are not publicly available due to the privacy policy.

## Supplementary Materials

The following supporting information can be downloaded at: https://www.mdpi.com/article/10.3390/jcm12072542/s1. Table S1: Demographics and clinical features of overall patients in “presentation-to-endoscopy” group by time frame. Table S2: Major outcomes of population in “presentation to endoscopy” group.
