# Supplementary material for: Defining Time in Acute Upper Gastrointestinal Bleeding: When Should We Start the Clock?
Source: J Clin Med. 2023 Mar 28;12(7):2542. doi: 10.3390/jcm12072542 (PMC10094998; doi:10.3390/jcm12072542)
Supplement: Supplementary file 1 [file jcm-12-02542-s001.zip › jcm-2245960-supplementary.pdf]

Supplementary files

Table S1. Demographics and clinical features of overall patients in "presentation-to-endoscopy" group by time frame

	**0/6h**	**6/12h**	**12/24h**	**≥24**	***p*-value**
	N=2072	N=520	N=399	N=175	
**Age, mean [± SD]**	67.1 [±15.8]	70.2 [±14.9]	70 [±16.1]	70.4 [±15.8]	<0.001
**Male gender, n(%)**	1429 (69)	348 (66.9)	269 (67.4)	107 (61.1)	
**ASA score, n(%)**					0.29
I	550 (26.5)	123 (23.7)	114 (28.6)	55 (31.4)	
III	674 (32.5)	175 (33.7)	120 (30.1)	60 (34.3)	
II	729 (35.2)	189 (36.3)	133 (33.3)	49 (28)	
IV	119 (5.7)	33 (6.3)	32 (8)	11 (6.3)	
**Syncope, yes, n (%)**	219 (10.6)	61 (11.7)	30 (7.5)	17 (9.7)	0.19
**Hematemesis, yes, n (%)**	1049 (50.6)	179 (34.4)	101 (25.3)	54 (30.9)	<0.001
**Melena, yes, n (%)**	1312 (63.3)	352 (67.7)	294 (73.7)	117 (66.9)	<0.001
**Hematemesis and melena, yes, n (%)**	385 (18.6)	57 (11)	34 (8.5)	17 (9.7)	<0.001
**Shock, yes, n (%)**	174 (8.4)	22 (4.2)	16 (4)	14 (8)	<0.001
**Rockall score, median (IQR)**	4 (3-5)	4 (3-5)	3.5 (2-5)	4 (3-5)	0.41
**GB score, median (IQR)**	7 (5-9)	8 (5-9)	7 (5-9)	8 (6-9)	0.80
**ABC score, median (IQR)**	5 (3-6)	5 (3-6)	5 (3-6)	4.5 (3-6)	0.74
**AIMS65 score, median (IQR)**	1 (1-2)	1 (1-2)	2 (1-2)	2 (1-2)	0.55
**PNED score, median (IQR)**	4 (2-6)	3 (1-5)	3 (1-5)	3 (1-5)	<0.001
**Hemoglobin level, gr/d**	9.1 (7.5-10.8)	8.9 (7.5-10.8)	8.5 (7.2-10.8)	8.5 (6.8-10)	0.006
**Bleeding source**					<0.001
**Non-variceal, n (%)**	1632 (78.8)	470 (90.4)	361 (90.5)	169 (96.6)	
**Variceal, n(%)**	440 (21.2)	50 (9.6)	38 (9.5)	6 (3.4)	

Table S2. Major outcomes of population in “presentation to endoscopy” group

	**0/6h**	**6/12h**	**12/24h**	**≥24h**	***p*-value**
	N=2072	N=520	N=399	N=175	
**Transfusion, yes (%)**	1211 (58.4)	302 (58.1)	258 (64.7)	114 (65.1)	0.044
**Rebleeding,yes (%)**	131 (6.3)	26 (5)	22 (5.5)	13 (7.4)	0.56
**Need for interventional radiology, yes (%)**	21 (1)	2 (0.4)	6 (1.5)	1 (0.6)	0.33
**Need for surgery, yes (%)**	64 (3.1)	13 (2.5)	17 (4.3)	8 (4.6)	0.34
**Mortality, yes (%)**	146 (7)	28 (5.4)	24 (6)	11 (6.3)	0.54

Table S3. Demographics and clinical features of overall patients in "symptoms-to-endoscopy" group by time frame

	**0/6h**	**6/12h**	**12/24h**	**≥24h**	***p*-value**
	N=1304	N=745	N=578	N=539	
**Age, mean [± SD]**	68.1 [±15.1]	68.3 [±15.6]	68.4 [±16.4]	67.9 [±16.7]	0.97
**Male gender, n(%)**	871 (66.8)	497 (66.7)	404 (69.9)	381 (70.7)	0.24
**ASA score, n(%)**					<0.001
I	297 (22.8)	185 (24.8)	172 (29.8)	188 (34.9)	
III	461 (35.4)	250 (33.6)	165 (28.5)	153 (28.4)	
II	455 (34.9)	276 (37)	203 (35.1)	166 (30.8)	
IV	91 (7)	34 (4.6)	38 (6.6)	32 (5.9)	
**Syncope, yes, n (%)**	140 (10.7)	84 (11.3)	57 (9.9)	46 (8.5)	0.40
**Hematemesis, yes, n (%)**	765 (58.7)	287 (38.5)	195 (33.7)	136 (25.2)	<0.001
**Melena, yes, n (%)**	717 (55)	504 (67.7)	430 (74.4)	424 (78.7)	<0.001
**Hematemesis and melena, yes, n (%)**	240 (18.4)	104 (14)	84 (14.5)	65 (12.1)	<0.001
**Shock, yes, n (%)**	118 (9)	40 (5.4)	31 (5.4)	37 (6.9)	0.003
**Rockall score, median (IQR)**	4 (3-5)	4 (2-5)	3 (2-5)	3.5 (2-5)	<0.001
**GB score, median (IQR)**	8 (5-9)	7 (4-9)	7 (5-9)	7 (6-9)	0.047
**ABC score, median (IQR)**	5 (3-7)	4 (3-6)	4 (3-6)	4 (3-6)	<0.001
**AIMS65 score, median (IQR)**	2 (1-2)	1 (1-2)	1 (1-2)	1 (1-2)	<0.001
**PNED score, median (IQR)**	4 (2-6)	3 (1-5)	3 (1-5)	3 (1-5)	<0.001
**Hemoglobin level, gr/d**	9 (7.5-10.7)	9.3 (7.7-11.1)	8.8 (7.4-11)	8.5 (6.8-10.4)	<0.001
**Bleeding source**					<0.001
**Non-variceal, n (%)**	971 (74.5)	647 (86.8)	508 (87.9)	506 (93.9)	
**Variceal, n(%)**	333 (25.5)	98 (13.2)	70 (12.1)	33 (6.1)	

Table S4. Clinical outcomes in the "symptoms-to-endoscopy" groups by time frame

	**0/6h**	**6/12h**	**12/24h**	**≥24h**	***p*-value**
	N=1304	N=745	N=578	N=539	
**Transfusion, yes (%)**	804 (61.7)	396 (53.2)	348 (60.2)	337 (62.5)	<0.001
**Rebleeding, yes (%)**	106 (8.1)	35 (4.7)	27 (4.7)	24 (4.5)	<0.001
**Need for interventional radiology, yes (%)**	20 (1.5)	6 (0.8)	3 (0.5)	1 (0.2)	0.024
**Need for surgery, yes (%)**	45 (3.5)	21 (2.8)	20 (3.5)	16 (3)	0.84
**Mortality, yes (%)**	117 (9)	38 (5.1)	31 (5.4)	23 (4.3)	<0.001

Table S5. Demographics and clinical features in the outpatients according to "presentation-to-endoscopy" group by time frame

	**0/6h**	**6/12h**	**12/24h**	**≥24h**	***p*-value**
	N=1730	N=378	N=319	N=124	
**Age, mean [± SD]**	66.4 [±15.9]	69.7 [±14.9]	69.7 [±16.8]	69 [±16.6]	<0.001
**Male gender, n(%)**	1207 (69.8)	259 (68.5)	220 (69)	76 (61.3)	0.27
**ASA score, n(%)**					0.55
I	495 (28.6)	101 (26.7)	98 (30.7)	46 (37.1)	
III	537 (31)	117 (31)	87 (27.3)	37 (29.8)	
II	617 (35.7)	140 (37)	116 (36.4)	36 (29)	
IV	81 (4.7)	20 (5.3)	18 (5.6)	5 (4)	
**Syncope, yes, n (%)**	195 (11.3)	55 (14.6)	26 (8.2)	14 (11.3)	0.07
**Hematemesis, yes, n (%)**	903 (52.2)	147 (38.9)	80 (25.1)	43 (34.7)	<0.001
**Melena, yes, n (%)**	1100 (63.6)	248 (65.6)	239 (74.9)	79 (63.7)	<0.001
**Hematemesis and melena, yes, n (%)**	342 (19.8)	48 (12.7)	31 (9.7)	14 (11.3)	<0.001
**Shock, yes, n (%)**	147 (8.5)	16 (4.2)	8 (2.5)	9 (7.3)	0.001
**Rockall score, median (IQR)**	4 (3-5)	4 (2-5)	3 (2-5)	4 (2-5)	0.27
**GB score, median (IQR)**	7 (5 -9)	8 (5 -9)	7 (5 -9)	8 (4 -9)	0.87
**ABC score, median (IQR)**	4 (3 -6)	4 (3 -6)	4 (3 -6)	4 (3 -5)	0.22
**AIMS65 score, median (IQR)**	1 (1 -2)	1 (1 -2)	1 (1 -2)	1.5 (1 -2)	0.47
**PNED score, median (IQR)**	4 (1 -6)	3 (1 -4)	3 (1 -5)	3 (1 -4.5)	<0.001
**Hemoglobin level, gr/d**	9.2 (7.5-10.9)	8.8 (7.5-10.8)	8.6 (7.2-10.8)	8.6 (6.6-10.4)	0.021
**Bleeding source**					<0.001
**Non-variceal, n (%)**	1333 (77.1)	335 (88.6)	293 (91.8)	119 (96)	
**Variceal, n(%)**	397 (22.9)	43 (11.4)	26 (8.2)	5 (4)	

Table S6. Outcomes in in the outpatients according to “presentation to endoscopy” group by time frame

	**0/6h**	**6/12h**	**12/24h**	**≥24h**	**p-value**
	N=1730	N=378	N=319	N=124	
**Transfusion, yes (%)**	1002 (57.9)	224 (59.3)	203 (63.6)	72 (58.1)	0.30
**Rebleeding, yes (%)**	98 (5.7)	17 (4.5)	14 (4.4)	5 (4)	0.60
**Need for interventional radiology, yes (%)**	14 (0.8)	2 (0.5)	2 (0.6)	1 (0.8)	0.94
**Need for surgery, yes (%)**	48 (2.8)	11 (2.9)	14 (4.4)	7 (5.6)	0.17
**Mortality, yes (%)**	97 (5.6)	19 (5)	11 (3.4)	3 (2.4)	0.21

Table S7. Demographics and clinical features of outpatients in the "symptoms-to-endoscopy" group

	0/6h	6/12h	12/24h	≥24h	*p*-value
	N=987	N=594	N=505	N=465	2
**Age, mean [± SD]**	67.2 [±15.2]	67.4 [±15.7]	68 [±16.9]	67.3 [±16.7]	0.82
**Male gender, n(%)**	670 (67.9)	403 (67.8)	355 (70.3)	334 (71.8)	0.38
**ASA score, n(%)**					0.005
I	248 (25.1)	169 (28.5)	154 (30.5)	169 (36.3)	
III	317 (32.1)	192 (32.3)	139 (27.5)	130 (28)	
II	368 (37.3)	209 (35.2)	185 (36.6)	147 (31.6)	
IV	54 (5.5)	24 (4)	27 (5.3)	19 (4.1)	
**Syncope, yes, n (%)**	119 (12.1)	75 (12.6)	52 (10.3)	44 (9.5)	0.30
**Hematemesis, yes, n (%)**	624 (63.2)	258 (43.4)	175 (34.7)	116 (24.9)	<0.001
**Melena, yes, n (%)**	538 (54.5)	386 (65)	373 (73.9)	369 (79.4)	<0.001
**Hematemesis and melena, yes, n (%)**	208 (21.1)	92 (15.5)	76 (15)	59 (12.7)	<0.001
**Shock, yes, n (%)**	94 (9.5)	34 (5.7)	25 (5)	27 (5.8)	0.001
**Rockall score, median (IQR)**	4 (3-5)	4 (2-5)	3 (2-5)	3 (2-5)	<0.001
**GB score, median (IQR)**	8 (5-9)	7 (4-9)	7 (4-9)	7 (6-9)	0.026
**ABC score, median (IQR)**	5 (3-6)	4 (3-6)	4 (3-6)	4 (3-5)	<0.001
**AIMS65 score, median (IQR)**	2 (1-2)	1 (1-2)	1 (1-2)	1 (1-2)	<0.001
**PNED score, median (IQR)**	4 (2-6)	3 (1-5)	3 (1-5)	2 (0-4)	<0.001
**Hemoglobin level, gr/d**	9.1 (7.5-10.7)	9.5 (7.8-11.2)	8.9 (7.4-11)	8.5 (6.8-10.5)	<0.001
**Bleeding source**					<0.001
**Non-variceal, n (%)**	701 (71)	502 (84.5)	439 (86.9)	438 (94.2)	
**Variceal, n(%)**	286 (29)	92 (15.5)	66 (13.1)	27 (5.8)	

Table S8. Outcomes in in the outpatients according to “symptoms-to-endoscopy” group

	**0/6h**	**6/12h**	**12/24h**	**≥24h**	***p*-value**
	N=987	N=594	N=505	N=465	
**Transfusion, yes (%)**	607 (61.5)	311 (52.4)	301 (59.6)	282 (60.6)	0.003
**Rebleeding, yes (%)**	68 (6.9)	29 (4.9)	19 (3.8)	18 (3.9)	0.023
**Need for interventional radiology, yes (%)**	11 (1.1)	6 (1)	2 (0.4)	0	0.082
**Need for surgery, yes (%)**	33 (3.3)	17 (2.9)	16 (3.2)	14 (3)	0.96
**Mortality, yes (%)**	70 (7.1)	29 (4.9)	18 (3.6)	13 (2.8)	0.001

Table S9. Concordance between the two time frames in the outpatient’s cohort (expressed as number of patients)

Symptoms onset to endoscopy group	Presentation to endoscopy group				
	**0-6hours**	**6-12hours**	**12-24hours**	**≥24hours**	**Total**
**0-6hours**	1028	13	4	5	1.050
**6-12hours**	381	213	20	4	618
**12-24hours**	191	128	198	8	525
**>24hours**	220	41	107	111	479
**Total**	1820	395	329	128	2672

Agreement= **77.4%;** Kappa = **0.41** SE= **0.01**

Expected agreement= **62%;** Z = **33.8,** Prob =**0.0000**

Table S10. Concordance between the two time frames in the inpatient’s cohort (expressed as number of patients)

Symptoms onset to endoscopy group	Presentation to endoscopy group				
	**0-6hours**	**6-12hours**	**12-24hours**	**≥24hours**	**Total**
**0-6hours**	201	9	24	20	254
**6-12hours**	24	94	6	3	127
**12-24hours**	4	17	27	5	53
**>24hours**	23	5	13	19	60
**Total**	252	125	70	47	494

Agreement = **81.4%;** Kappa=**0.47** SE=**0.03**

Expected agreement= **64%;** Z=**14,** Prob =**0.0000**

Table S11. Effect of "presentation-to-endoscopy" timing on mortality in outpatients

	Odds Ratio	S. E.	z	P>|z|	[95% C.I.]
**0-6hours**					
**6-12hours**	0.83	0.21	-0.71	0.477	0.50-1.38
**12-24hours**	0.68	0.20	-1.29	0.197	0.38-1.22
**>24hours**	0.40	0.23	-1.56	0.118	0.12-1.27
**_cons**	0.06	0.01	-27.76	0.000	0.05-0.07

Table S12. Effect of "symptoms to endoscopy" timing on mortality in outpatients

	Odds Ratio	S. E.	z	P>|z|	[95% C.I.]
**0-6hours**					
**6-12hours**	0.69	0.15	-1.71	0.087	0.45- 1.06
**12-24hours**	0.51	0.13	-2.58	0.010	0.31-0.85
**>24hours**	0.36	0.11	-3.32	0.001	0.20-0.66
**_cons**	0.08	0.01	-21.41	0.000	0.06-0.10
